# Confocal laser endomicroscope with distal MEMS scanner for real-time histopathology

**DOI:** 10.1038/s41598-022-24210-9

**Published:** 2022-11-23

**Authors:** Miki Lee, Gaoming Li, Haijun Li, Xiyu Duan, Mayur B. Birla, Tse-Shao Chang, Danielle K. Turgeon, Kenn R. Oldham, Thomas D. Wang

**Affiliations:** 1 Department of Internal Medicine, University of Michigan, Ann Arbor, 48109 USA; 2 Department of Mechanical Engineering, University of Michigan, Ann Arbor, 48109 USA; 3 Department of Biomedical Engineering, University of Michigan, Ann Arbor, 48109 USA

**Keywords:** Gastroenterology, Engineering, Optics and photonics

## Abstract

Confocal laser endomicroscopy is an emerging methodology to perform real time optical biopsy. Fluorescence images with histology-like quality can be collected instantaneously from the epithelium of hollow organs. Currently, scanning is performed at the proximal end of probe-based instruments used routinely in the clinic, and flexibility to control the focus is limited. We demonstrate use of a parametric resonance scanner packaged in the distal end of the endomicroscope to perform high speed lateral deflections. An aperture was etched in the center of the reflector to fold the optical path. This design reduced the dimensions of the instrument to 2.4 mm diameter and 10 mm length, allowing for forward passage through the working channel of a standard medical endoscope. A compact lens assembly provides lateral and axial resolution of 1.1 and 13.6 μm, respectively. A working distance of 0 μm and field-of-view of 250 μm × 250 μm was achieved at frame rates up to 20 Hz. Excitation at 488 nm was delivered to excite fluorescein, an FDA-approved dye, to generate high tissue contrast. The endomicroscope was reprocessed using a clinically-approved sterilization method for 18 cycles without failure. Fluorescence images were collected during routine colonoscopy from normal colonic mucosa, tubular adenomas, hyperplastic polyps, ulcerative colitis, and Crohn’s colitis. Individual cells, including colonocytes, goblet cells, and inflammatory cells, could be identified. Mucosal features, such as crypt structures, crypt lumens, and lamina propria, could be distinguished. This instrument has potential to be used as an accessory during routine medical endoscopy.

## Introduction

Confocal laser endomicroscopy is an emerging imaging modality that is being developed for clinical use as an accessory to routine medical endoscopy^1–3^. These flexible fiber-coupled instruments can be used to identify diseases in the epithelium that lines hollow organs, such as colon. This thin layer of tissue is highly metabolically active, and is the origin of many disease processes, such as cancer, infection, and inflammation. Endomicroscopy can achieve sub-cellular resolution to provide in vivo images with “histology-like” quality in real time to guide physicians in making clinical decisions. Physical biopsy of tissue incurs a risk for bleeding and perforation. Often, either too many or too few biopsies are collected. Each specimen resected increases the cost of the procedure. Several days are required to process the specimen for evaluation by a pathologist. Patients often experience anxiety while waiting several days for the pathology results to become available. By comparison, other clinical imaging methods, such as MRI, CT, PET, SPECT, and ultrasound, lack the spatial resolution and temporal speeds required to visualize epithelial processes with sub-cellular resolution in vivo in real time.

A probe-based instrument (Cellvizio) is currently used routinely in the clinic to perform “optical biopsy.” The design is based on a spatially coherent fiber bundle that collects and transmits fluorescence images^4^. The cores of individual fibers serve as “pinholes” to spatially filter out-of-focus light to achieve sub-cellular resolution. Scanning is performed at the proximal end using large, bulky galvos. This location limits the ability of the instrument to control the focus. Adequate staging of early epithelial cancers requires visualization below the tissue surface to assess invasion to determine appropriate therapy. Fluorescein, an FDA approved contrast agent, is injected intravenously to highlight the structural features of the epithelium. These endomicroscopes have dimensions <2.4 mm in diameter, and can be passed forward easily through the biopsy channel of standard medical endoscopes. This flexibility allows for broad clinical use, and is independent of the endoscope manufacturer. Numerous clinical studies have been performed using this imaging instrument, including in esophagus, stomach, colon, and oral cavity, for early cancer detection. Imaging protocols have been established, and safety for this procedure has been determined.

Microelectromechanical systems (MEMS) is a powerful technology to design and fabricate miniature scanning mechanisms for use in the distal end of endomicroscopes. This location (versus proximal) offers much greater flexibility to control the position of the focus^5,6^. In addition to lateral deflections, distal mechanisms can perform axial, post-objective, and random access scanning. These functions allow for more comprehensive interrogation of the epithelium, including vertical cross-sectional imaging^7^, aberration-free scanning over a large field-of-view (FOV)^8^, and improved performance in user-defined sub-regions^9^, respectively. MEMS addresses the considerable challenges posed by the limited space available in the distal tip of the instrument to package the scanning mechanism. By comparison with bulky galvos, MEMS provides superior performance in a small form factor with high speed and low power. Simple fabrication processes can be scaled up for mass manufacture at low cost. A number of MEMS designs have been reported previously^10–12^. None have been developed sufficiently to perform real-time in vivo imaging through the working channel of a medical endoscope to enable broad clinical translation. Here, we aim to demonstrate the use of a MEMS scanner in the distal tip of an endomicroscope to collect in vivo images in human subjects during routine clinical endoscopy.

## Results

### Endomicroscope

**Figure 1:**
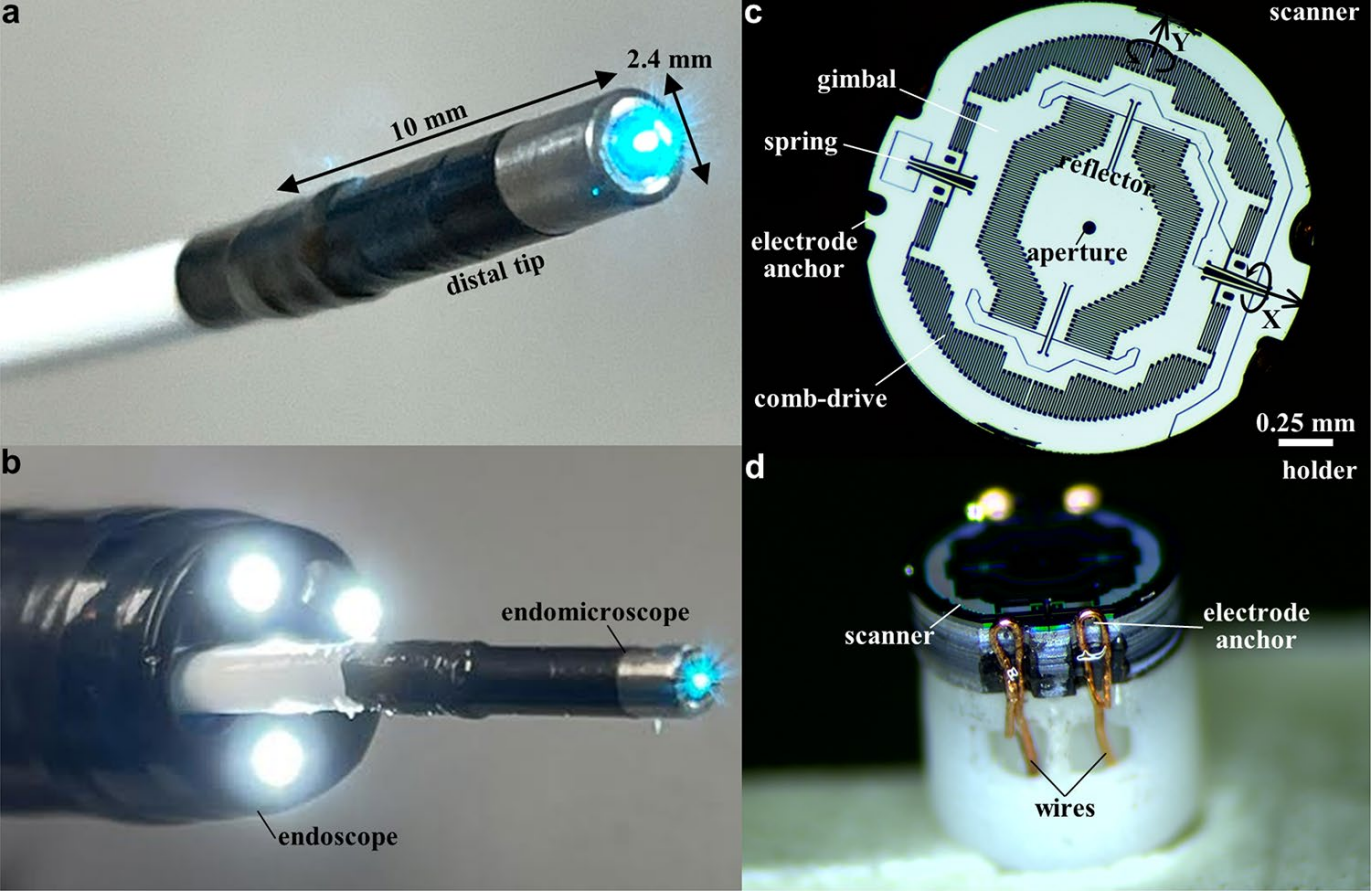
Confocal laser endomicroscope (CLE) and MEMS scanner. Photo is shown of (**a**) the packaged instrument with rigid distal tip dimensions of 2.4 mm in diameter and 10 mm in length, and (**b**) forward passage through the working channel of a standard medical endoscope (Olympus CF-HQ190L). (**c**) Front view of the scanner shows a reflector with a 50 μm diameter central aperture to allow excitation beam to pass. The scanner is mounted on a gimbal driven by a set of orthogonal comb-drive actuators. The resonance frequencies of this device are determined by the dimensions of the torsional springs. (**d**) Side-view of the scanner shows scanner mounted on the holder with wires to electrode anchors that provide points of connection for the drive signals and power.

A fiber-coupled instrument was developed using MEMS scanner in the distal tip to collect real time fluorescence images in vivo with histology-like features. A single mode fiber (SMF) was enclosed in a flexible polymer tube, and delivered excitation at λ_ex_ = 488 nm. This configuration shortens the length of the distal tip, and allows for forward passage through the working channel of standard medical endoscopes. A ferrule was used to center the optical components. The lenses were designed to achieve near diffraction-limited resolution on axis with a numerical aperture (NA) = 0.41 and working distance = 0 μm^13^. Precision spacers were fabricated to accurately align the optics^14^. The scanner was packaged into an endomicroscope with a rigid distal tip of 2.4 mm diameter and 10 mm length (Fig. 1a). These dimensions allow for clinical use as an accessory during endoscopy (Fig. 1b). The maximum laser power incident on the tissue was 2 mW.

### Scanner

The scanning mechanism consisted of a reflector mounted on a gimbal driven by a set of orthogonal comb-drive actuators to deflect the beam laterally (XY-plane) in a Lissajous pattern (Fig. 1c). A 50 μm diameter aperture was etched in the center to pass the excitation beam. The scanner was driven near the resonance frequencies of the structure, which could be tuned by modifying the dimensions of the torsional springs. Electrode anchors were etched around the periphery of the device to provide connection points for power and drive signal (Fig. 1d).

### Imaging system

The imaging system was arranged on a portable cart that could be wheeled into the procedure room. A graphical user interface was developed to support users with minimal technical expertise, such as physicians and nurses. The scanner drive frequency, beam shape pattern, and image FOV was checked manually.

The total length of the endomicroscope was ~4 m to allow the instrument to pass completely through the working channel (1.68 m) of a standard medical endoscope with extra length for maneuverability. At the proximal end of the endomicroscope, the SMF and wires were terminated by connectors, that were inserted into the fiber and wire ports of the base station. This unit contained the laser, optical filter block, high-voltage amplifiers, and photomultiplier tube (PMT) detector. The amplifiers delivered power and drive signals to the scanner. The optical filter block coupled laser excitation into the SMF, and delivered fluorescence to the PMT.

The endomicroscope was reprocessed after each clinical procedure using the STERRAD sterilization process, and survived up to 18 cycles without failure. For the OPA solution, no signs of damage were found after over 10 disinfection cycles. The results for OPA were better than that with STERRAD, and suggests that the lifespan of the endomicroscopes can be extended with high-level disinfection rather than sterilization for reprocessing.

### Imaging performance

**Figure 2:**
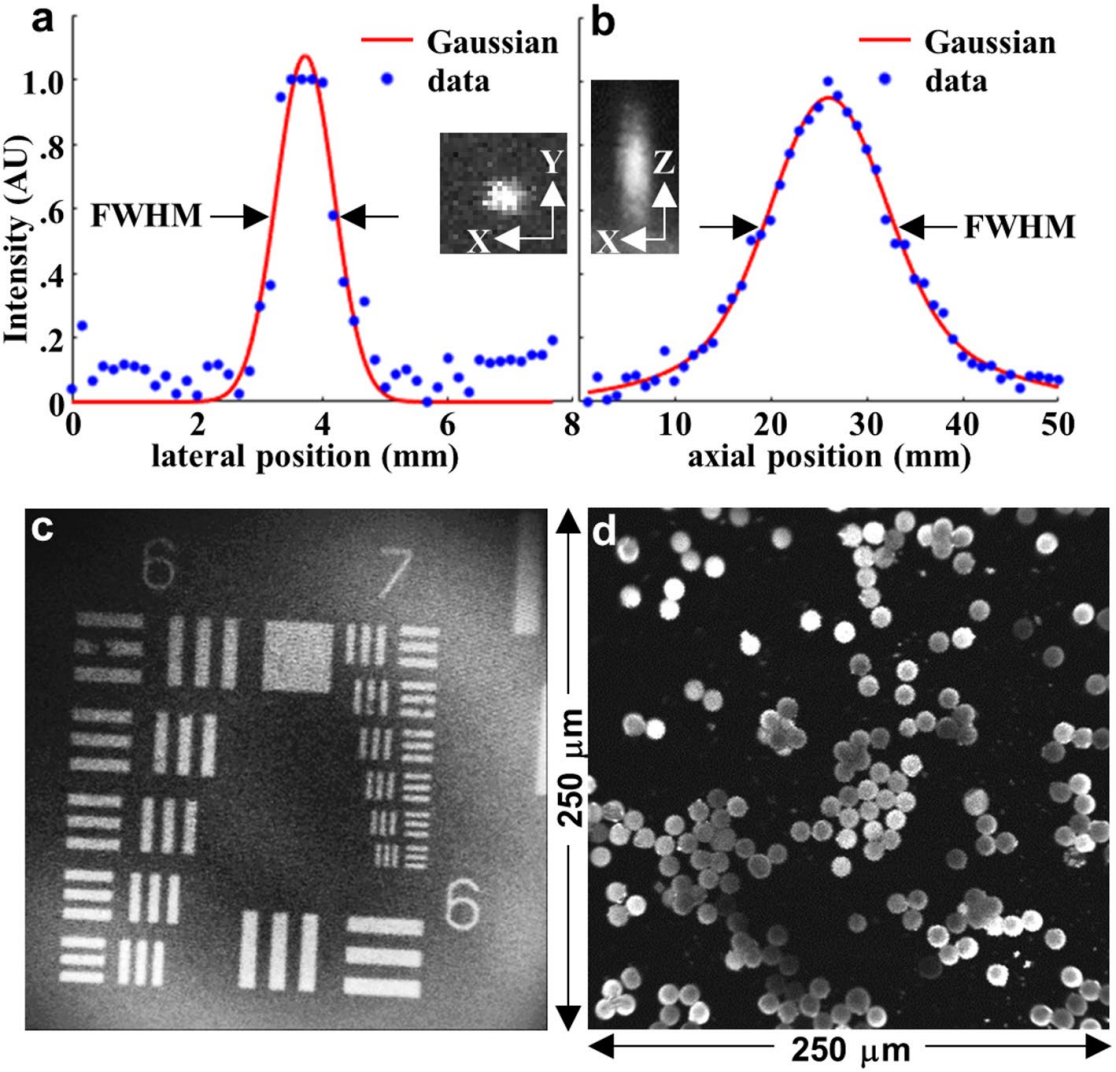
Image parameters. (**a)** Lateral and (**b**) axial resolution of focusing optics was characterized by a point spread function (PSF) measured using 0.1 μm diameter fluorescent microspheres. A full-width-half-maximum (FWHM) of 1.1 and 13.6 μm, respectively, was measured. Inset: expanded view of single microspheres in the lateral (XY) and axial (XZ) directions are shown. (**c**) A fluorescence image collected from a standard (USAF 1951) target bars (red oval) shows that group 7–6 can be clearly resolved. (**d**) An image of dispersed 10 μm diameter fluorescent microspheres demonstrates an image FOV of 250 μm × 250 μm. PSFs in (**a, b**) were plotted using MATLAB R2019a (https://www.mathworks.com/). (**c, d**) Fluorescence images were collected using LabVIEW 2021 (https://www.ni.com/).

Image resolution was determined by the point spread function using 0.1 μm diameter fluorescent beads. A full-width-at-half-maximum (FWHM) of 1.1 and 13.6 μm was measured for the lateral and axial resolution, respectively (Fig. 2a,b).

A fluorescence image of a standard resolution target clearly distinguished the set of bars in group 7-6 to support high lateral resolution (Fig. 2c). A field-of-view (FOV) of 250 μm × 250 μm was determined from an image of 10 μm diameter fluorescent beads dispersed over a glass cover slip (Fig. 2d).

### System software

Automated methods for PMT gain control and phase correction were implemented in the clinical imaging system to mitigate motion artifact from the endoscope, colon peristalsis, and patient breathing. The image reconstruction and processing algorithms have been described previously^14,15^. The PMT gain was adjusted using a proportional-integral (PI) controller to prevent intensity saturation^16^. The system reads a maximum pixel intensity per frame, computes the proportional and integral response, and determines a value for the PMT gain to provide pixel intensities within an acceptable range.

### Image processing

**Figure 3:**
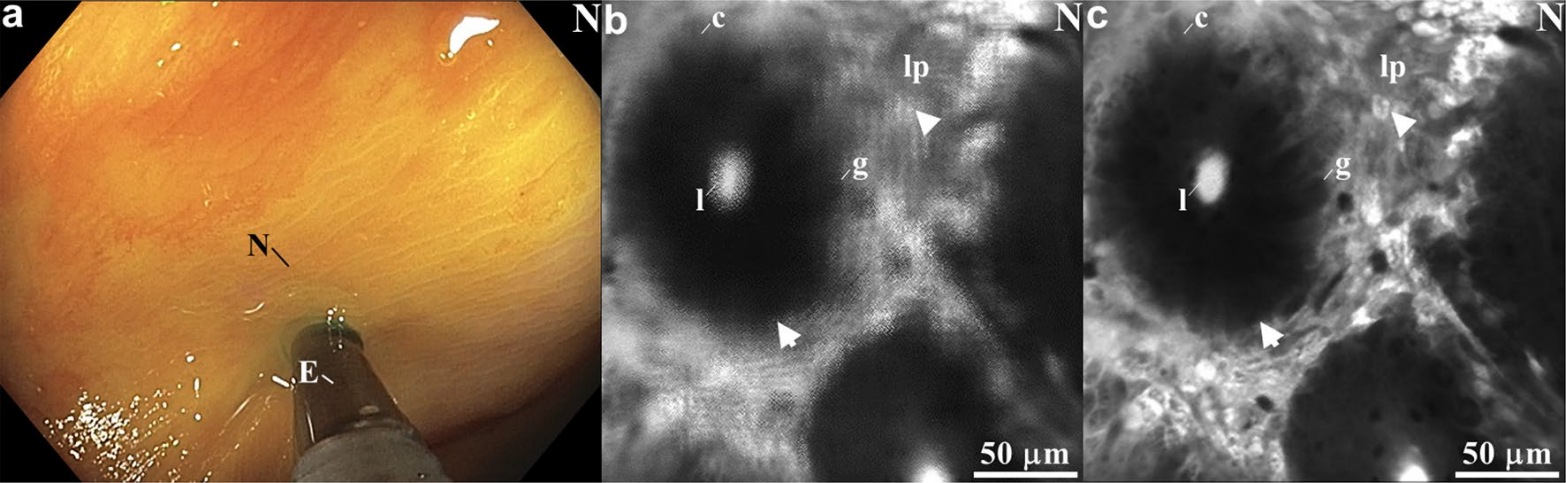
Image processing. (**a**) Wide-field endoscopic image shows endomicroscope (E) placed in contact with normal (N) colonic mucosa for collection of fluorescence images in vivo following administration of fluorescein. (**b**) Drift in phase of the X and Y-axes during scanning may result in blurring from misregistered pixels. Large phase shifts were applied to the original image for demonstration. (**c**) After post-processed phase correction, mucosal details can be appreciated, including crypt structure (arrow) with central lumen (l) surrounded by lamina propria (lp). Individual cells can be distinguished, including colonocytes (c), goblet cells (g), and inflammatory cells (arrowhead). See Supplementary Video 1. (**b, c**) Images were processed using LabVIEW 2021.

During in vivo imaging, a mismatch in phase between the scanner motion and the driving signal may result in blurry images. This effect may occur from temperature variations with the instrument inside the human body. A white light image shows the endomicroscope placed in contact with normal colonic mucosa in vivo (Fig. 3a). Blurring from misregistered pixels can be seen in the unprocessed image from normal colonic mucosa (Fig. 3b). After processing using the correct phase and contrast adjustment, sub-cellular features in the mucosa could be distinguished (Fig. 3c). In Supplementary Information, the raw confocal and the real-time processed images are provided in Fig. S1, and the image reconstruction parameters used for the real-time and post-processing are provided in Tables S1 and S2.

### In vivo confocal images

Confocal fluorescence images were collected in vivo from several disease conditions in the colon to demonstrate the broad clinical applicability of this instrument. Wide-field imaging was first performed using white light illumination to identify grossly abnormal appearing mucosa. The endomicroscope was then passed forward through the working channel of the colonoscope and placed in contact with the mucosa.

**Figure 4:**
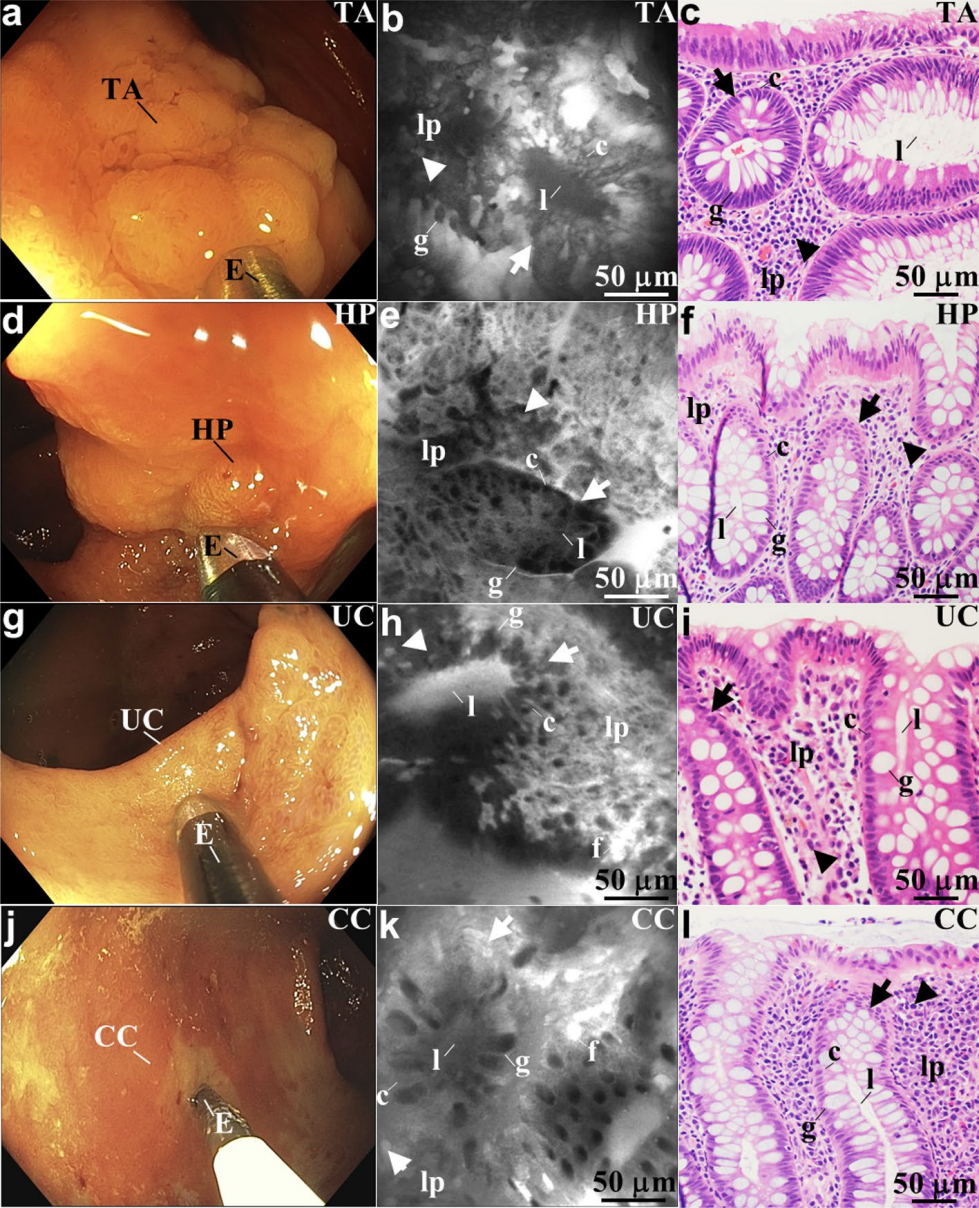
In vivo images of colonic mucosa. Representative white light endoscopy, confocal endomicroscope, and histology (H&E) images are shown for (**a-c**) adenoma, (**d-f**) hyperplastic polyp, (**g-i**) ulcerative colitis, and (**j-l**) Crohn’s colitis. (**b**) Confocal image collected with the endomicroscope (E) in vivo from a tubular adenoma (TA) is shown. This pre-malignant lesion shows a loss of normal crypt (arrow) architecture, distorted crypt lumens (l), and crowded lamina propria (lp). Colonocytes (c), goblet cells (g), and inflammatory cells (arrowhead) can also be identified. See Supplementary Video 2. (**e**) Confocal image collected in vivo from a hyperplastic polyp (HP) is shown. This benign lesion shows a stellate crypt (arrow) structure, slit-shaped crypt lumens (l), and irregular lamina propria (lp). Colonocytes (c), few goblet cells (g), and inflammatory cells (arrowhead) can also be identified. See Supplementary Video 3. (**h**) Confocal image collected in vivo from ulcerative colitis (UC) is shown. This inflammatory condition shows distorted crypt (arrows) architecture with prominent goblet cells (g). Fluorescein (f) plumes extrude from the epithelium reflecting increased vascular permeability. Plenty of inflammatory cells (arrowheads) can be seen in the lamina propria (lp). See Supplementary Video 4. (**k**) Confocal image collected in vivo from patchy areas of Crohn’s colitis (CC) is shown. This inflammatory condition shows distorted crypt (arrows) architecture with prominent goblet cells (g). Fluorescein (f) plumes extrude from the epithelium reflecting increased vascular permeability. Plenty of inflammatory cells (arrowheads) can be seen in the lamina propria (lp). See Supplementary Video 5. (**b, e, h, k**) Images were processed using LabVIEW 2021.

Wide-field endoscopy, confocal endomicroscopy, and histology (H&E) images are shown for colonic neoplasia, including tubular adenoma and hyperplastic polyp. The tubular adenoma shows a loss of normal crypt architecture, decreased goblet cell size, distorted crypt lumens, and crowded lamina propria (Fig. 4a–c). The hyperplastic polyp shows a stellate crypt structure, few goblet cells, slit-shaped crypt lumens, and irregular lamina propria (Fig. 4d–f).

A similar set of images are shown for colonic inflammation, including ulcerative colitis (UC) (Fig. 4g–i), and Crohn’s colitis (Fig. 4j–l). An inflammatory reaction can be appreciated characterized by distorted crypt architecture with prominent goblet cells. Fluorescein extrudes from the epithelium, a reflection of increased vascular permeability. Numerous inflammatory cells can be seen in the lamina propria.

## Discussion

We have demonstrated clinical use of a flexible fiber-coupled confocal laser endomicroscope using a MEMS scanner located in the distal end to collect in vivo images. At resonant frequencies, frame rates up to 20 Hz were achieved with a Lissajous scan pattern with high density to mitigate motion artifact. The optical path was folded to allow the beam to expand and generate an NA sufficient to achieve 1.1 μm lateral resolution. Fluorescence images were collected with histology-like quality during routine colonoscopy from normal colonic mucosa, tubular adenomas, hyperplastic polyps, ulcerative colitis, and Crohn’s colitis. Individual cells, including colonocytes, goblet cells, and inflammatory cells, could be identified. Mucosal features, such as crypt structures, crypt lumens, and lamina propria, could be distinguished. Precision apparatus were microfabricated to provide accurate alignment of the individual optical and mechanical components within a 2.4 mm diameter and 10 mm length instrument. The optical design adequately reduced the length of the rigid distal tip to allow for forward passage through a working channel with standard dimensions (3.2 mm diameter) in medical endoscopes. This instrument can thus be broadly used by community physicians regardless of manufacturer. Excitation at λ_ex_ = 488 nm was delivered to excite fluorescein, an FDA-approved dye, to generate high contrast. The instrument was reprocessed using clinically approved sterilization methods for 18 cycles without failure.

Two other instrument designs have been demonstrated in the clinic. The Cellvizio (Mauna Kea Technologies) is a probe-based confocal laser endomicroscope (pCLE) that uses a multi-mode coherent optical fiber bundle to collect and transmit fluorescence images^1^. A galvo located in the base station performs lateral scanning at the proximal end. Optical sections are collected in the horizontal (XY) plane at depths from 0 to 70 μm. A set of miniprobes with diameters from 0.91 (19 G needle) to 5 mm are available. Lateral resolution between 1 and 3.5 μm has been achieved. Images are collected at frame rates between 9 and 12 Hz with a FOV between 240 and 600 μm in one dimension. This platform has been used clinically in a number of applications, including in the bile duct, bladder, colon, esophagus, lung, and pancreas. Optiscan Pty Ltd developed an endoscope-based confocal laser endomicroscope (eCLE) with the scanning mechanism integrated into the insertion tube (distal end) of a specialty endoscope (EC-3870K, Pentax Precision Instrument)^17^. A single mode fiber was used to perform optical sectioning, and lateral scanning was performed using a cantilever mechanism by a resonant tuning fork. A shape memory alloy (Nitinol) actuator was used to generate axial displacement. The overall diameter of the confocal module was 5 mm. A GRIN lens with NA = 0.6 was used for focusing. Horizontal images were collected with a lateral and axial resolution of 0.7 and 7 μm, respectively, at frame rates of 0.8-1.6 Hz with a FOV of 500 μm × 500 μm.

We demonstrated the use of a MEMS scanner located in the distal tip to collect in vivo fluorescence images with sub-cellular resolution from human subjects via a medical endoscope. Fluorescence provides high image contrast, and ligands that bind to cell surface targets can be labeled with fluorophores to provide molecular properties to improve disease diagnosis^18^. Other optical methods are being developed for in vivo endomicroscopy. OCT uses the short coherence length from a broadband light source to collect images in the vertical plane with depths >1 mm^19^. However, this low contrast methodology is based on collection of back scattered light, and image resolution is limited by speckle artifact. Photoacoustic endomicroscopy generates in vivo images based on a rapid thermoelastic expansion in tissue following absorption of a laser pulse, and generates an acoustic wave^20^. This approach has demonstrated imaging depths >1 cm in human colon in vivo to monitor therapy. Contrast is generated primarily from hemoglobin in the vasculature. Multi-photon endomicroscopy generates high contrast fluorescence images when two or more NIR photons arrive at a tissue biomolecule simultaneously^21^. This approach can achieve imaging depths >1 mm with low phototoxicity. High intensity femtosecond laser pulses are required, and this modality has not been demonstrated clinically during endoscopy.

In this prototype, the scanner performed lateral deflection only, thus optical sections were displayed in the horizontal plane (XY). This device was able to achieve frame rates (20 Hz) higher by comparison to the galvos in the Cellvizio system (12 Hz). The frame rate was increased to mitigate motion artifact and decreased to increase signal. High speeds and automated algorithms will be required to mitigate large motion artifacts from endoscope movement, respiratory motion, and gut peristalsis. Parametric resonance scanners have been demonstrated to achieve axial displacements over several hundred microns^22^. Images can be collected in vertical plane (XZ), perpendicular to the mucosal surface, to provide the same view as that of histology (H&E). The scanner can be placed in the post-objective position where the illumination beam is incident along the main optical axis to reduce sensitivity to aberrations^8^. A near diffraction-limited focal volume can be deflected over an arbitrarily large FOV. Random access scanning can be performed to deflect the reflector in user-defined positions^9^. The FOV can be narrowed to highlight arbitrary image sub-regions to improve the signal-to-noise ratio, contrast, and frame rate. Scanners can be batch fabricated using simple processes. Hundreds of devices can be produced on each silicon wafer to scale up production for mass manufacture and broad dissemination at low cost.

The folded optical path reduced the dimensions of the rigid distal tip to permit seamless use of the endomicroscope as an accessory during routine colonoscopy. In the fluorescence images shown, sub-cellular mucosal features could be visualized to distinguish tubular adenomas (pre-malignant) from hyperplastic polyps (benign). These results demonstrate potential for endomicroscopy to reduce unnecessary biopsies^23^. Overall procedure-related complications may be decreased, surveillance intervals may be optimized, and histological analysis of insignificant lesions may be minimized. We also demonstrated in vivo images from patients with inflammatory bowel disease, including ulcerative colitis (UC) and Crohn’s colitis. Conventional white light colonoscopy provides a macroscopic appearance of the mucosal surface with limited ability to accurately assess mucosal healing. Endomicroscopy may be used in vivo to assess the effectiveness of biologic therapies, such as anti-TNF antibodies^24^. Accurate in vivo assessment may also reduce or prevent disease relapse and complications, such as surgery, and improve quality of life. In the clinical study, there were no serious adverse reactions reported related to the in vivo use of the endomicroscope with the fluorescein^25^. The laser power on the mucosal surface was limited to <2 mW to minimize risk for thermal injury and meet the FDA requirements for non-significant risk^26^ per 21 CFR 812.

The design of this instrument can be modified to improve imaging performance. Custom optics can be used to reduce spherical aberrations, improve image resolution, and increase working distance. The SIL can be tailored to better match the index of refraction of tissue (~1.4) to improve light coupling. The drive frequency can be tuned to increase the lateral deflection angle of the scanner to enlarge the image FOV. Automated methods can be implemented to remove image frames with significant motion to mitigate this effect. A field programmable gate array (FPGA) with high-speed data acquisition will be implemented to achieve high-performance, full-frame correction in real time. For greater clinical utility, automated methods should correct for the phase shifts and motion artifact to interpret images in real-time. A monolithic 3-axis parametric resonance scanner can be implemented to introduce axial scanning^22^. These devices have been developed to achieve unprecedented vertical displacement >400 µm by tuning the drive frequency in a regime that features mixed softening/stiffening dynamics^27^. In the future, vertical cross-sectional imaging may be useful for staging early cancer (T1a). A capacitive sensing circuit can be implemented to track the scanner motion and correct phase shifts^28^. Automated phase calibration using a sensing circuit may replace manual calibration prior to instrument use. The reliability of the instrument can be improved with use of more robust methods to seal the instrument to increase the number of reprocessing cycles. MEMS technology promises to accelerate the use of endomicroscopes to visualize the epithelium of hollow organs to diagnose disease and monitor therapy in a minimally invasive manner. With further development, this emerging imaging methodology may become a low-cost solution for use as an accessory to medical endoscopy to provide instantaneous histology and may eventually replace conventional pathological analysis.

## Methods

### Optical design

**Figure 5:**
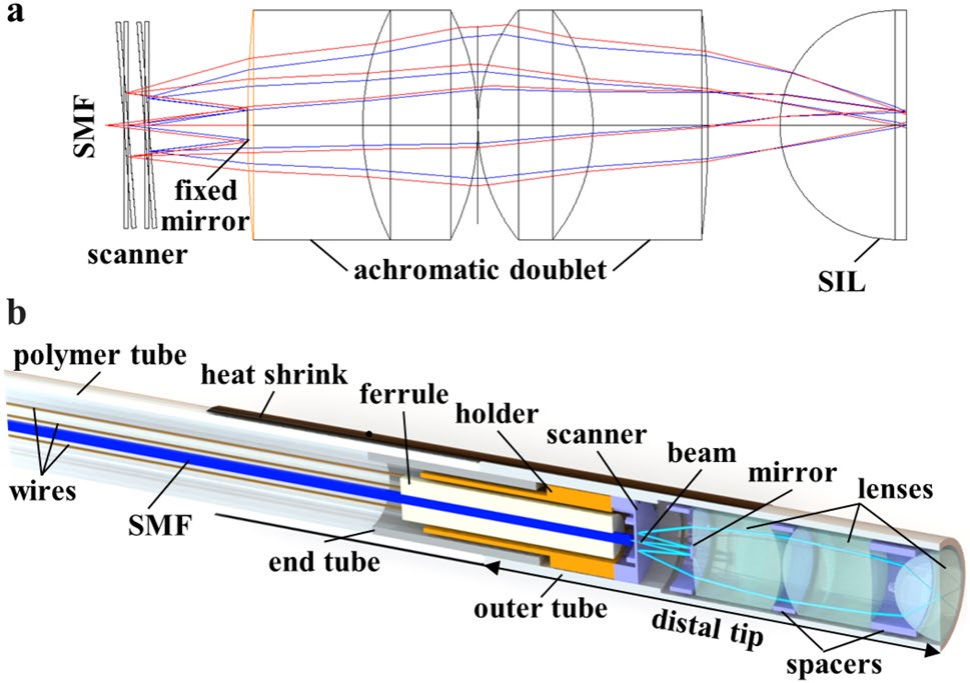
Folded optical path and endomicroscope packaging. (**a**) The excitation beam exits the SMF, and passes through the center aperture of the scanner. The beam expands and reflects off a fixed circular mirror back toward the scanner for lateral deflections. The focusing optics consists of a pair of achromatic doublets and a solid immersion (half-ball) lens that provides contact with the mucosal surface. ZEMAX 2013 (https://www.zemax.com/) was used for the optical design and ray-trace simulation. (**b**) Packaging arrangement for the individual components of instrument, including single mode fiber (SMF), scanner, mirror, and lenses, is shown. Solidworks 2016 (https://www.solidworks.com/) was used for the 3D modeling of the endomicroscope packaging.

Ray-trace simulations were performed using ZEMAX (ver 2013) optical design software to define the parameters for the focusing optics. The design criteria included near diffraction-limited resolution on axis, working distance = 0 μm, and field-of-view (FOV) greater than 250×250 μm^2^. A single mode fiber (SMF) was used to deliver excitation at λ_ex_ = 488 nm. Achromatic doublets were used to mitigate chromatic dispersion for fluorescence collection (Fig. 5a). The beam was delivered through a SMF with a mode-field diameter of 3.5 μm, and passes the center of the reflector with 50 μm diameter aperture without truncation. A solid immersion (half-ball) lens with high refractive index (n = 2.03) was used to minimize spherical aberrations from the incident beam and provide comprehensive contact with the mucosal surface. The focusing optics provides a total NA = 0.41, where NA = nsinα, n is the index of refraction of tissue, and α is the maximum convergence angle of the beam. The diffraction limit for lateral and axial resolution is 0.44 and 6.65 μm, respectively, using values of NA = 0.41, λ = 488 nm, and n = 1.33^13^. Only commercially available lenses with outer diameter (OD) ≤ 2 mm were considered. The optical path was folded whereby the beam exiting the SMF passed through a central aperture in the scanner, and was reflected backwards by a fixed mirror (0.29 mm diameter). This configuration shortens the length of the rigid distal tip to facilitate forward passage of the endomicroscope through a standard (3.2 mm diameter) working channel in medical endoscopes. This capability allows for seamless use as an accessory during routine endoscopy.

### Endomicroscope

A SMF with 3.5 μm mode-field diameter at 488 nm (#460HP, Thorlabs) was used to serve as a “pinhole” to spatially filter out-of-focus light (Fig. 5b). The SMF was enclosed in a flexible polymer tube (#Pebax 72D, Nordson MEDICAL). A length of ~4 meters was used to provide sufficient distance between the patient and imaging system. A pair of 2 mm diameter MgF_2_ coated achromatic doublets (#65568, #65567, Edmund Optics) and an uncoated 2 mm diameter half-ball lens (#90858, Edmund Optics) were used to focus the beam and collect fluorescence. A stainless steel end tube (4 mm length, 2.0 mm OD, 1.6 mm ID) was inserted in between the polymer and outer tubes to isolate the scanner from vibrations. A medical-grade adhesive was applied to seal the instrument from bodily fluids and during reprocessing. A heat shrink tube was used to protect the interface.

### Scanner

A compact scanner was fabricated based on the principle of parametric resonance^14^. A 50 μm aperture was etched in the center of the reflector to pass the excitation beam. The expanded beam was deflected laterally in orthogonal directions (XY-plane) in a Lissajous pattern using a set of orthogonal comb-drive actuators. A data acquisition board (#DAQ PCI-6115, NI) was used to create the analog waveform to drive the scanner. Power was provided by high voltage amplifiers (#PDm200, PiezoDrive) via fine electrical wires (#B4421241, MWS Wire Industries). Wiring was performed on electrode anchors. The scanner was driven at frequencies near 15 kHz (fast-axis) and 4 kHz (slow-axis) to achieve a FOV up to 250 μm × 250 μm. Videos could be collected at frame rates of 10, 16, or 20 Hz. These frame rates were used to match the repetition rate of the Lissajous scan pattern, which depends on the value of the scanner X- and Y- driving frequencies^29^. Details on the tradeoffs among the frame rate, pixel resolution, and scan pattern density are provided in our previous work^14^.

### Imaging system

**Figure 6:**
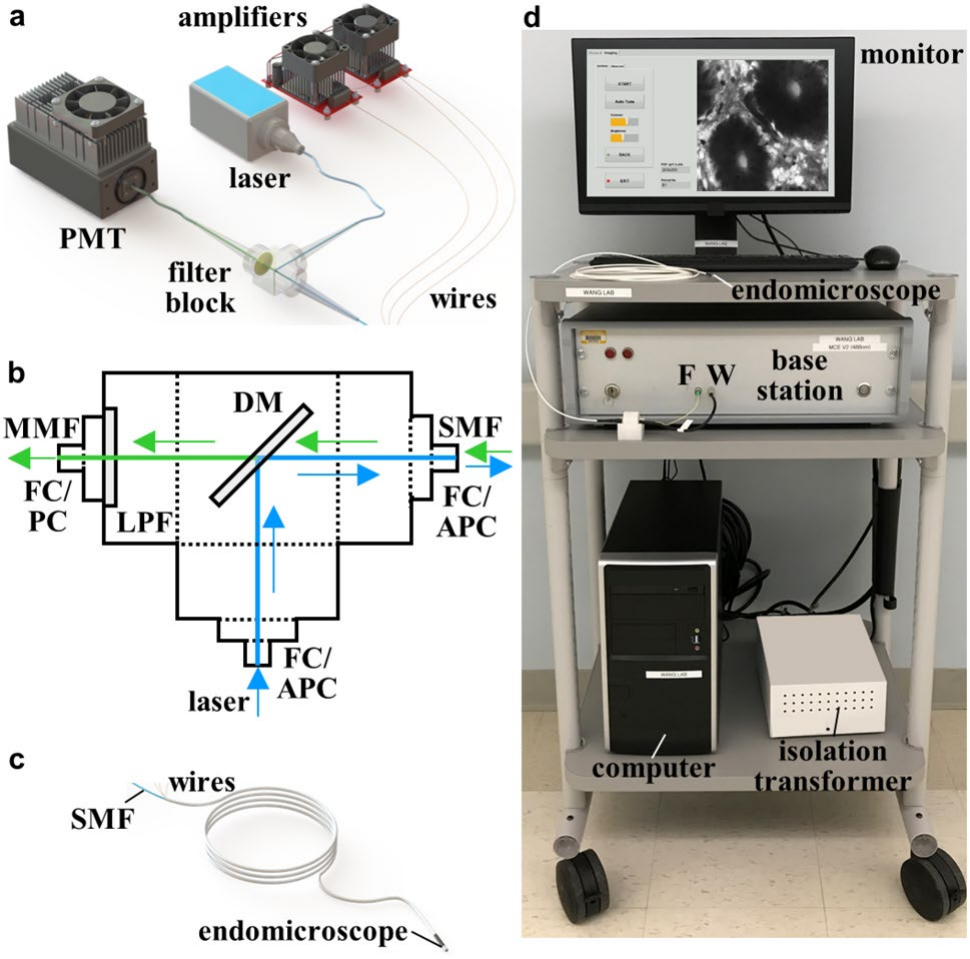
Imaging system. (**a**) The PMT, laser, and amplifiers are contained within the base station. (**b**) In the filter block, the laser (blue) delivers excitation via a fiber pigtail via a FC/APC connector. The beam is deflected by a dichroic mirror (DM) into a single mode fiber (SMF) via a second FC/APC connector. Fluorescence (green) passes through the DM and a long pass filter (LPF) to the PMT via a multimode fiber (MMF). (**c**) The proximal end of the endomicroscope connects to the fiber (F) and wire (W) ports of the base station. (**d**) The endomicroscope, monitor, base station, computer, and isolation transformer are contained on a portable cart. **(a, c)** Solidworks 2016 was used for 3D modelling of the imaging system assembly and the endomicroscope.

A solid-state laser (#OBIS 488 LS, Coherent) delivered λ_ex_ = 488 nm to excite fluorescein for image contrast (Fig. 6a). The fiber pigtail was coupled to an optical filter block via an FC/APC connector (1.82 dB loss) (Fig. 6b). The beam was deflected by a dichroic mirror (#WDM-12P-111-488/500:600, Oz Optics) into the SMF via another FC/APC connector. The power incident on tissue was limited to a maximum of 2 mW to meet the FDA requirements for non-significant risk per 21 CFR 812. Fluorescence passed through the dichroic mirror and a long-pass filter (#BLP01-488R, Semrock). A ~1 m long multimode fiber with 50 μm core diameter was used to transmit fluorescence via a FC/PC connector to the photomultiplier tube (PMT) detector (#H7422-40, Hamamatsu). Fluorescence signal was amplified by a high-speed current amplifier (#59-179, Edmund Optics). Custom software (LabVIEW 2021, NI) was developed to perform real-time data acquisition and image processing. The laser power and PMT gain settings were determined by a microcontroller (#Arduino UNO, Arduino) using a custom-printed circuit board. The SMF and wires were terminated by connectors, and inserted into the fiber (F) and wire (W) ports of the base station (Fig. 6c). The imaging system was contained on a portable cart (Fig. 6d). An isolation transformer was used to limit the leakage current to <500 μA.

### Imaging performance

The lateral and axial resolution of the focusing optics was measured from the point-spread-function of 0.1 μm diameter fluorescent microspheres (#F8803, Thermo Fisher Scientific). Images were collected by translating the microspheres in the horizontal and vertical direction in 1 μm increments using a linear stage (#M-562-XYZ, DM-13, Newport). Images were stacked using ImageJ2 to obtain cross-sectional images from the microspheres.

### System software

**Figure 7:**
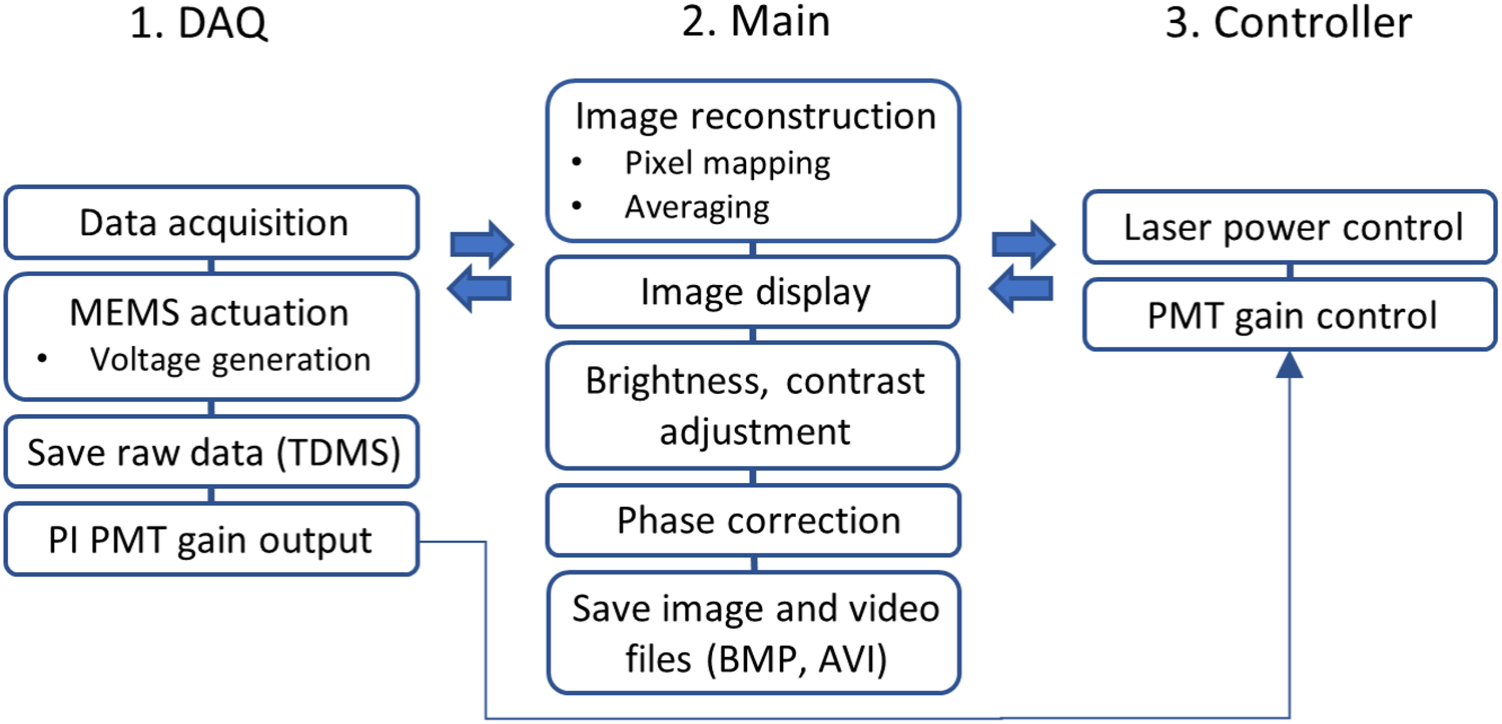
System software architecture. The user interface includes modules for (1) data acquisition (DAQ), (2) main panel, and (3) controller panel. The programs run simultaneously and communicate with each other via message queuing. Key – MEMS: microelectromechanical systems, TDMS: technical data management streaming, PI: proportional-integral, PMT: photomultiplier tube. Image and video files are saved in BMP and AVI format, respectively.

Custom software (LabVIEW 2021, NI) was developed to perform real-time data acquisition and image processing. An overview of the routines used for system operation is shown in Fig. 7. The user interface consists of data acquisition (DAQ), main, and controller panels. The DAQ panel communicates with the main panel to acquire and save raw data, provides input for the user-defined data acquisition settings, and controls the scanner actuation parameters. The main panel allows the user to select the desired configuration to use the endomicroscope, including drive signals to the scanner, video frame rate, and data acquisition parameters. This panel also allows users to display and control image brightness and contrast. Using raw data as the input, an algorithm computes the optimal gain setting for the PMT, and automatically adjusts this parameter using a proportional-integral (PI) based feedback control system^16^. The controller panel communicates with both the main and DAQ panels to control the laser power and PMT gain.

### Image processing

A phase correction algorithm was used to compute the variance in image pixel intensities at various values for phase to determine the largest value to sharpen the image^15^. For real-time correction, the phase was swept over a range of ±2.86° with a relatively large step size of 0.286° to reduce computation time. Also, a partial region of the image with a smaller number of samples was used to further reduce the computation time from 7.5 s (1 M samples) to 1.88 s (250 K samples) per image frame at 10 Hz. These input parameters were chosen to provide sufficient image quality with minimal delay during in vivo imaging. Real-time images and videos are recorded in BMP and AVI formats, respectively. Raw data was saved in Technical Data Management Streaming (TMDS) format.

The in vivo images were post-processed to enhance quality using LabVIEW 2021. The phase correction algorithm had limited accuracy when used during in vivo imaging because of the long computation times required. Only a limited image area and number of samples were used. Moreover, the algorithm was less effective for images with either motion artifact or low contrast, and led to phase miscalculations^30^. Individual frames with high contrast and without motion artifact were manually selected to fine tune the phase with a phase sweep range of ±0.75° and a step size of 0.01°. The full image area (e.g., 1 M samples for images recorded at 10 Hz) was used. Details on the image parameters used for the real-time and post-processing are summarized in Table S2. After phase correction, image noise was further reduced using a median filter. Brightness and contrast were further enhanced through histogram stretching and gamma correction^31^.

### In vivo confocal images

The clinical study was approved by the Institutional Review Board at Michigan Medicine, and was performed in the Medical Procedures Unit. The study was registered online at ClinicalTrials.gov (NCT03220711, date of registration: 18/07/2017). Inclusion criteria included patients (ages from 18 years to 100 years) previously scheduled for routine colonoscopy, at increased risk for colorectal cancer, and with a history of inflammatory bowel disease. Informed consent was obtained from each subject who agreed to participate. Exclusion criteria included patients who were pregnant, had known allergy to fluorescein, or were on active chemotherapy or radiation therapy. Consecutive patients scheduled for routine colonoscopy were recruited for this study, and represented the population seen at Michigan Medicine. The study was performed in accordance with the Declaration of Helsinki.

Prior to the procedure, the endomicroscope was calibrated using 10 μm diameter fluorescent beads (#F8836, Thermo Fisher Scientific) fixed in a silicone mold. A translucent silicone sealant (#RTV108, Momentive) was poured into an 8 cm^3^ 3D printed plastic mold. Aqueous fluorescent beads were dropped on top of the silicone and were left until the aqueous media dried out.

A standard medical colonoscope (Olympus, CF-HQ190L) was used to examine the entire colon using white light illumination. Once the endoscopist identified a region suspicious for disease, the site was rinsed with 5-10 mL of 5% acetic acid followed by sterile water to remove mucus and debris. A 5 mL dose of 5 mg/mL fluorescein (Alcon, Fluorescite) was either injected intravenously or topically sprayed onto the mucosa using a standard cannula (M00530860, Boston Scientific) passed through the working channel.

An irrigator was used to wash away any excess dye or debris on the mucosal surface. The spray catheter was removed, and the endomicroscope was then passed through the working channel to collect in vivo images. The distal tip was positioned onto the target region using wide-field endoscopy for guidance. The total time used to collect confocal images was <10 min. White light endoscopy videos were processed with the Olympus EVIS EXERA III (CLV-190) imaging system and recorded using Elgato HD video recorder. Endomicroscopy videos were recorded and saved using LabVIEW 2021. After completion of imaging, the endomicroscope was removed, and either a biopsy forceps or snare was used to resect the tissues imaged. The tissues were processed for routine histology (H&E), and evaluated by an expert GI pathologist (H.D.A.). The spectral properties of fluorescein were confirmed using a spectrometer (USB2000+, Ocean Optics) as shown in Fig. S2.

### Endoscope sterilization

**Figure 8:**
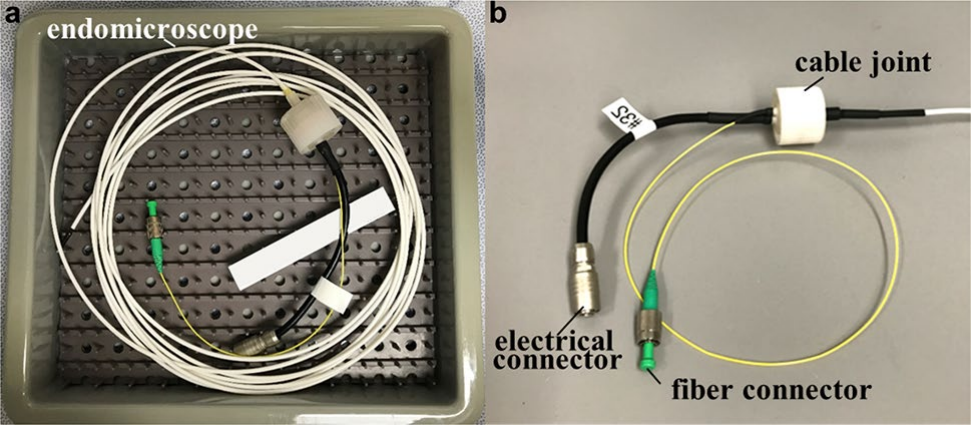
Instrument reprocessing. (**a**) The endomicroscope was placed in a tray after each procedure for sterilization with the STERRAD reprocessing process. (**b**) The SMF and wires were terminated with fiber and electrical connectors, respectively, that were capped prior to reprocessing.

The endomicroscope were sterilized after each use in human subjects (Fig. 8). The cleaning procedure was performed under guidance and with approval of the Department of Infection Control and Epidemiology and the Central Sterile Reprocessing Department at Michigan Medicine. Prior to the study, the instruments were tested and validated for sterilization by Advanced Sterilization Products (ASP, Johnson & Johnson), a commercial entity that provides infection prevention and sterilization validation services.

The endomicroscope was reprocessed using the following steps: (1) The endomicroscope was wiped from the proximal to the distal end with a lint-free cloth soaked in enzymatic detergent; (2) The instrument was immersed in an enzymatic detergent solution for 3 min, rinsed with water, and dried with a lint-free cloth. The electrical and fiber connectors were capped, and left out of the solution; (3) The endomicroscope was wrapped and placed in an instrument tray for sterilization using STERRAD 100NX, a reprocessing system that uses hydrogen peroxide gas plasma technology at relatively low temperature and in a low moisture environment for a standard cycle of 47 min.

## Supplementary Information

Supplementary Information 1.

Supplementary Video 1.

Supplementary Video 2.

Supplementary Video 3.

Supplementary Video 4.

Supplementary Video 5.

## Data Availability

The datasets used and/or analyzed during the current study are available from the corresponding author on reasonable request.

## References

[1] Pilonis ND, Januszewicz W, di Pietro M (2022). Confocal laser endomicroscopy in gastro-intestinal endoscopy: Technical aspects and clinical applications. Transl. Gastroenterol. Hepatol..

[2] Al-Mansour MR (2021). SAGES TAVAC safety and efficacy analysis confocal laser endomicroscopy. Surg. Endosc..

[3] Fugazza A (2016). Confocal laser endomicroscopy in gastrointestinal and pancreatobiliary diseases: A systematic review and meta-analysis. Biomed. Res. Int..

[4] Al-Gubory KH (2019). Shedding light on fibered confocal fluorescence microscopy: Applications in biomedical imaging and therapies. J. Biophotonics..

[5] Shao Y, Dickensheets DL (2005). MOEMS 3-D scan mirror for single-point control of beam deflection and focus. J. Microlith. Microfab. Microsyst..

[6] Murakami K (ed. Ürey H.). A miniature confocal optical scanning microscope for endoscopes. Proceedings SPIE 5721, MOEMS Display and Imaging Systems III. **5721**, 119–131 (MOEMS-MEMS Micro and Nanofabrication, San Jose, CA, 2005).

[7] Li G (2017). Visualizing epithelial expression in vertical and horizontal planes with dual axes confocal endomicroscope using compact distal scanner. IEEE Trans. Med. Imaging..

[8] Duan X (2017). Three-dimensional side-view endomicroscope for tracking individual cells in vivo. Biomed. Opt. Express..

[9] Ashida Y, Hamann S, Landry J, Solgaard O (2020). Conjugated MEMS phased arrays for large field of view random access scanning. IEEE Photonics Technol. Lett..

[10] Seo YH, Hwang K, Jeong KH (2018). 1.65 mm diameter forward-viewing confocal endomicroscopic catheter using a flip-chip bonded electrothermal MEMS fiber scanner. Opt. Express..

[11] Hwang K (2020). Handheld endomicroscope using a fiber-optic harmonograph enables real-time and in vivo confocal imaging of living cell morphology and capillary perfusion. Microsyst. Nanoeng..

[12] Jeon J (2022). Handheld laser scanning microscope catheter for real-time and in vivo confocal microscopy using a high definition high frame rate Lissajous MEMS mirror. Biomed. Opt. Express..

[13] Corle, T.R. & Kino, G.S. (eds) in Confocal Scanning Optical Microscopy and Related Imaging Systems 1st edn, Ch.2–3 (Academic Press, 1996).

[14] Li G (2020). Ultra-compact microsystems-based confocal endomicroscope. IEEE Trans. Med. Imaging..

[15] Birla M (2021). Image processing metrics for phase identification of a multiaxis MEMS scanner used in single pixel imaging. IEEE ASME Trans. Mechatron..

[16] Astrom, K.J. & Murray, R.M. (eds) in Feedback Systems: An Introduction for Scientists and Engineers 4th edn, Ch.10 (Princeton University Press, 2008).

[17] Piyawattanametha, W. & Wang, T.D. in In Vivo Clinical Imaging and Diagnosis 1st edn, (ed. Tunnell J.W.) Pt.1, Ch.2 (McGraw Hill Professional, 2011).

[18] Sturm MB (2013). Targeted endoscopic imaging of Barrett's neoplasia with specific fluorescent-labeled peptide: First in-human results. Sci. Trans. Med..

[19] Gora MJ, Suter MJ, Tearney GJ, Li X (2017). Endoscopic optical coherence tomography: Technologies and clinical applications. Biomed. Opt. Express..

[20] Leng X (2021). Assessing rectal cancer treatment response using coregistered endorectal photoacoustic and US imaging paired with deep learning. Radiology.

[21] Kučikas, V., Werner, M.P., Schmitz-Rode, T., Louradour, F. & van Zandvoort, M.A.M.J. Two-photon endoscopy: State of the art and perspectives. *Mol. Imaging Biol*. (2021). 10.1007/s11307-021-01665-2 PMC9971078 34779969

[22] Li H (2016). Integrated monolithic 3D MEMS scanner for switchable real time vertical/horizontal cross-sectional imaging. Opt. Express..

[23] Morarasu S, Haroon M, Morarasu BC (2019). Colon biopsies: Benefit or burden?. J. Med. Life..

[24] Atreya R, Neumann H, Neufert C (2014). In vivo imaging using fluorescent antibodies to tumor necrosis factor predicts therapeutic response in Crohn's disease. Nat. Med..

[25] Wallace MB (2010). The safety of intravenous fluorescein for confocal laser endomicroscopy in the gastrointestinal tract. Aliment. Pharmacol. Ther..

[26] Holbein ME, Berglund JP (2012). Understanding food and drug administration regulatory requirements for an investigational device exemption for sponsor-investigators. J. Investig. Med..

[27] Li H (2019). Large-displacement vertical electrostatic microactuator dynamics using duty-cycled softening/stiffening parametric resonance. J. Micromech. Microeng..

[28] Chen Y (2020). Motion estimation for a compact electrostatic microscanner via shared driving and sensing electrodes in endomicroscopy. IEEE/ASME Trans. Mechatron..

[29] Hwang K, Seo YH, Ahn J, Kim P, Jeong KH (2017). Frequency selection rule for high definition and high frame rate Lissajous scanning. Sci. Rep..

[30] Yu, J. (ed.) et al. Estimating Perturbations to Laser Position on Tissue for Lissajous Scanning in Endomicroscopy. IEEE/ASME International Conference on Advanced Intelligent Mechatronics. 1561–1566 (IEEE, Boston, 2020).

[31] Dougherty, G (ed.) in Digital Image Processing for Medical Applications 1st edn, Ch.5 (Cambridge University Press, 2009).

