# Supplementary material for: Confocal laser endomicroscope with distal MEMS scanner for real-time histopathology
Source: Sci Rep. 2022 Nov 23;12:20155. doi: 10.1038/s41598-022-24210-9 (PMC9684518; doi:10.1038/s41598-022-24210-9)
Supplement: Supplementary file 1 — Supplementary Information 1. [file 41598_2022_24210_MOESM1_ESM.docx]

**Supplementary Information**

**Confocal Laser Endomicroscope with Distal MEMS Scanner for Real-Time Histopathology**

**Miki Lee**1,+**, Gaoming Li**1,+**, Haijun Li**1**, Xiyu Duan**1**, Mayur B. Birla**2**, Tse-Shao Chang**2**, Danielle K. Turgeon**1**, Kenn R. Oldham**2**, and Thomas D. Wang**1,2,3,*

1University of Michigan, Department of Internal Medicine, Ann Arbor, 48109, USA

2University of Michigan, Department of Mechanical Engineering, Ann Arbor, 48109, USA

3University of Michigan, Department of Biomedical Engineering, Ann Arbor, 48109, USA

*thomaswa@umich.edu

+these authors contributed equally to this work

**Supplementary Tables**

| Supplementary Video | | Image [pixels] | Frame rate [Hz] | MEMS drive frequency  (fast-axis, slow-axis) [kHz] |
| --- | --- | --- | --- | --- |
| 1 | normal | 400×400 | 10 | 40.84, 9.96 |
| 2 | tubular adenoma | 400×400 | 10 | 30.44, 7.24 |
| 3 | hyperplastic polyp | 400×400 | 16 | 40.864, 10.016 |
| 4 | ulcerative colitis | 400×400 | 20 | 40.84, 9.96 |
| 5 | Crohn’s colitis | 400×400 | 20 | 40.84, 9.96 |

**Table S1**. Image reconstruction and display parameters used to collect in vivo images are shown.

| Supplementary Video | Frame rate [Hz] | MEMS phase (fast-axis, slow-axis) [deg] | | Scan pattern density [%] | | Maximum number of intensities averaged per pixel | | Phase computation time [s] | |
| --- | --- | --- | --- | --- | --- | --- | --- | --- | --- |
|  |  | Real-time | Post-processed | Real-time | Post-processed | Real-time | Post-processed | Real-time | Post-processed |
| 1 | 10 | 189.27, 14.66 | 189.17, 14.37 | 94.2 | 95.8 | 512 | 512 | 1.88 | 55.53 |
| 2 | 10 | 22.84, 29.54 | 22.71, 28.84 | 92.7 | 94.3 | 512 | 512 | 1.88 | 54.66 |
| 3 | 16 | 184.86, 7.66 | 185.06, 7.06 | 89.6 | 94.6 | 318 | 317 | 1.88 | 34.64 |
| 4 | 20 | 186.76, 10.13 | 185.96, 11.33 | 95.7 | 95.3 | 254 | 256 | 1.88 | 27.85 |
| 5 | 20 | 185.96, 12.66 | 185.96, 12.19 | 93.4 | 96.4 | 254 | 254 | 1.88 | 27.80 |

**Table S2**. Parameters for real-time processed versus post-processed images are summarized.

**Supplementary Figures**


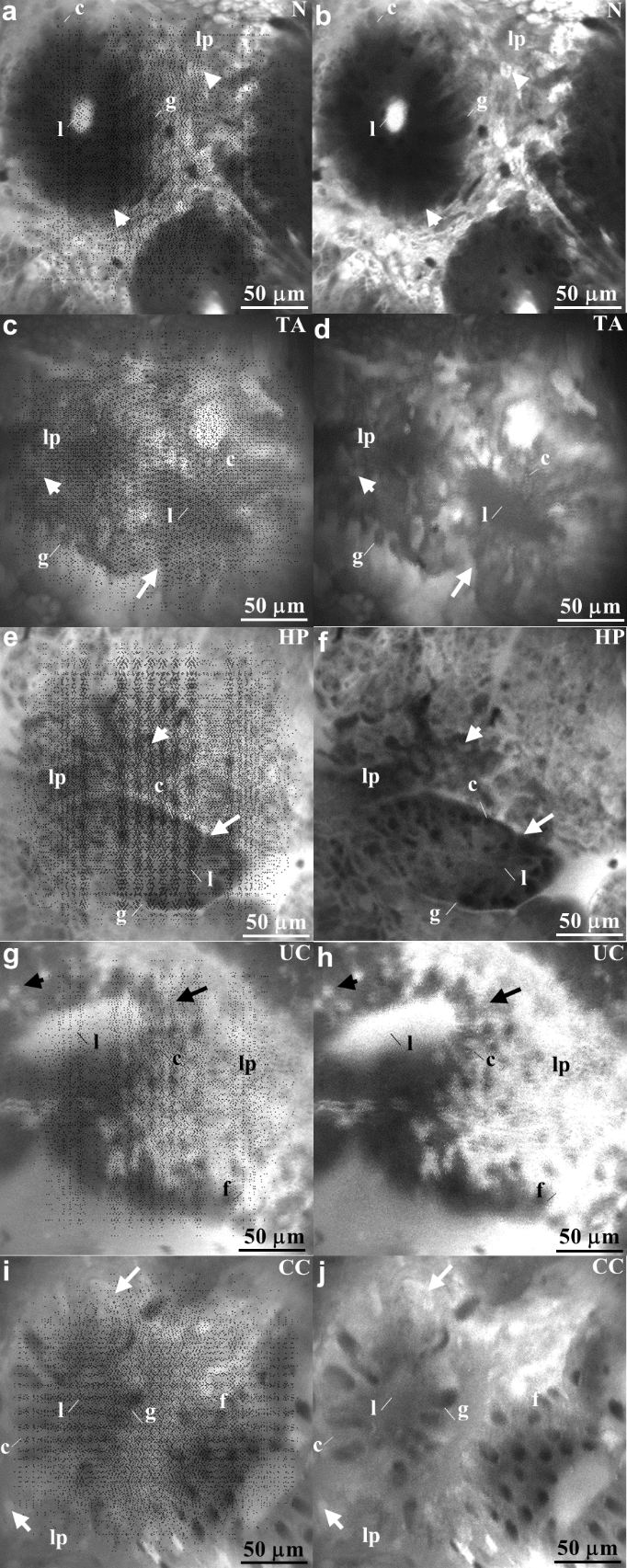


**Figure** **S1**. In vivo confocal endomicroscope images are shown for (**a-b**) normal (N), (**c-d**) tubular adenoma (TA), (**e-f**) hyperplastic polyp (HP), (**g-h**) ulcerative colitis (UC), and (**i-j**) Crohn’s colitis (CC). The raw confocal images collected in real-time (**a, c, e, g, i**) are shown without filling in the missing pixels and adjusting brightness or contrast. The real-time processed confocal images (**b, d, f, h, j**) are shown with slight phase shifts and under- or over-saturation. (**a-j**) Images were processed using LabVIEW 2021.


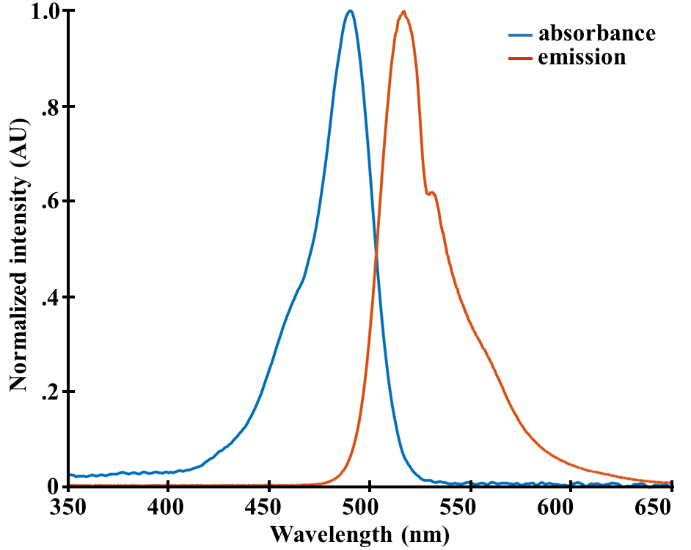


**Figure S2.** Spectral properties of fluorescein. The absorption and emission spectra for fluorescein (5 mg/mL) excited at 𝜆_ex_ = 488 nm shows peak fluorescence intensity at 𝜆_em_ = 516.6 nm. Spectra were collected using OceanView 2.0 software (https://www.oceaninsight.com/) and plotted using MATLAB R2019a.

**Supplementary Videos**

**Supplementary Video 1.** Post-processed confocal images of human normal colonic mucosa in the sigmoid captured at 10 fps. The scale bar at the bottom right corner represents 50 μm.

**Supplementary Video 2.** Post-processed confocal images of human tubular adenoma in the ascending colon captured at 10 fps. The scale bar at the bottom right corner represents 50 μm.

**Supplementary Video 3.** Post-processed confocal images of human hyperplastic colonic mucosa in the rectum captured at 16 fps. The scale bar at the bottom right corner represents 50 μm.

**Supplementary Video 4.** Post-processed confocal images of human ulcerative colitis in the cecum captured at 20 fps. The scale bar at the bottom right corner represents 50 μm.

**Supplementary Video 5.** Post-processed confocal images of human Crohn’s colitis in the rectum captured at 20 fps. The scale bar at the bottom right corner represents 50 μm.
